# Changes in Caregivers Lifestyle after Severe Acquired Brain Injury: A Preliminary Investigation

**DOI:** 10.1155/2018/2824081

**Published:** 2018-07-03

**Authors:** M. D'Ippolito, M. Aloisi, E. Azicnuda, D. Silvestro, M. Giustini, F. Verni, R. Formisano, U. Bivona

**Affiliations:** ^1^Post-Coma Unit, IRCCS Fondazione Santa Lucia, Rome, Italy; ^2^Department of Psychology, “La Sapienza” University of Rome, Italy; ^3^Ph.D. Program in Behavioral Neuroscience, Sapienza University of Rome, Italy; ^4^Environmental and Social Epidemiology Unit, National Institute of Health, Rome, Italy; ^5^Neuropsychological Rehabilitation Service, IRCCS Fondazione Santa Lucia, Rome, Italy; ^6^Child and Adolescent Neuropsychiatric Unit, University Hospital, Bari, Italy

## Abstract

**Introduction:**

Severe acquired brain injury (sABI) is considered the most common cause of death and disability worldwide. sABI patients are supported by their caregivers who often exhibit high rates of psychological distress, mood disorders, and changes in relationship dynamics and family roles.

**Objectives:**

To explore lifestyle changes of caregivers of sABI patients during the postacute rehabilitation, by investigating possible differences between primary and secondary caregivers. Primary caregivers spend most of the time with the patient, providing daily care and taking most responsibility for the day-to-day decisions, while secondary caregivers are those who provide additional support.

**Methods:**

Three hundred forty-seven caregivers of sABI patients were asked to fill in an unpublished self-report questionnaire to explore their possible lifestyles changes.

**Results:**

A statistically significant difference was found between primary and secondary caregivers in time spent in informal caregiving (p<0.001). The primary caregivers reduced all leisure activities compared to secondary carers (p<0.05).

**Conclusions:**

By comparing the percentage of leisure activities performed by caregivers* before* and* after *the patient's sABI onset, all caregivers showed high percentages of changes in lifestyle and habits, even though primary caregivers reported more negative lifestyle changes than secondary caregivers. Further studies are needed to investigate needs and burden experienced by caregivers of sABI patients during the postacute rehabilitation phase, also in relation to the patients' outcome, to address support interventions for them and improve their quality of life.

## 1. Introduction

Acquired brain injury (ABI) can occur after birth caused by either traumatic (TBI) (motor vehicle accidents, falls, assault, surgery, etc.) or nontraumatic brain injury (stroke, brain tumors, hypoxia, poisoning, etc.).

According to the Medical Disability Society (1988), an ABI is defined as* severe* (sABI) when coma lasts at least 6 hours [1, 2], and it is considered as the most common cause of death and disability worldwide, as it usually results in cognitive, physical, emotional, or behavioral impairments that lead to permanent or temporary changes in functioning and can severely impact the survivor's quality of life (QoL) [3, 4]. More specifically, severe TBI (sTBI) is the main cause of persistent long-term disability, since patients with TBI often show executive functioning, attention, information processing, memory, learning, and language deficits [5, 6]. Furthermore psychosocial sequelae, such as decreased life satisfaction and lower perceived social support [7, 8], may lead to substance abuse, anxiety, and depression [9, 10]. These cognitive, emotional, and behavioral changes can also cause family burden [11, 12], defined as the extent to which caregivers feel that their emotional or physical health, social life, and financial status have suffered as a result of caring for their relatives [13]. In fact, a large number of patients with sABI are supported by their family members, since they need continuous support and assistance in activities of daily living [14]. Caregivers are often deeply involved in the patient's management providing extraordinary and demanding care, and they often exhibit high rates of psychological distress [15], mood disorders, decreased QoL [16], and reduced personal independence [17]. They report putting themselves second to provide intensive support to the relative and, especially when the illness is in the critical phase, family members describe that their whole existence is entirely focused on the patient, feeling a limitation on their personal freedom [18, 19]. There is an ongoing issue about how carers define themselves (e.g., “caregivers”, “parents”, or “supporters”), but this does not affect the indisputable strain of the role [20]. An* informal caregiver* is defined as a person who, voluntary and without payment, provides care and support to someone in his/her family or social network with physical, mental, or psychiatric disabilities [21]. The relationship with the care receiver, as well as the health problem, has an impact on the amount of time spent in caregiving and, in general, those caring for their child or spouse spent more hours providing care than others [22]. Caregiving scenarios may be also differentiated in terms of the intensity of caregiving duties and the duration of the caregiving relationship [23]. In particular,* primary* caregivers generally provide most of cares to the patient, and take most responsibility for the daily decisions, being engaged in different areas of assistance (e.g., personal care, financial assistance, and housekeeping) [24] and becoming at greatest risk of poor psychosocial outcome [25];* secondary* caregivers do not have primary responsibility for the patient care [23], even though they may show high levels of psychological distress [25]. More specifically, a* primary* caregiver is defined as the individual who spends most of the time with the patient [26], while the* secondary* caregiver provides additional support (e.g., siblings, neighbors, or friends) [27, 28].* Secondary* caregivers are typically younger than* primary* ones [27, 29], and the care provided ranges from intensive personal care (i.e., eating, dressing, and toileting) to instrumental tasks (e.g., using the telephone, shopping, and taking medications) and emotional support [27–29].

As Jennings [30] noted, “the entire kinship system shakes” after a brain damage, and changes in relationship dynamics and family roles have been shown in some ABI studies [31, 32]. Indeed, postinjury perceived* role changes* are thought to be more problematic for spouses than parents [33], since spouses often perceive a loss of role symmetry in their relationship, observing a change from being a romantic partner to assuming, for example, the role of parent, due to helping the loved one with personal care tasks, such as dressing and toileting. Also changes in sexual behaviors, often shown after sTBI [34, 35], have a significant impact on the QoL of both TBI patients and their partners [36, 37]. Indeed, a recent study by Bivona and colleagues [38] revealed a reduction in desire and frequency of sexual intercourse in all male sTBI patients and their partners, and this reduced quality of sexual life seems to be more related to a relationship dysfunction than a sexual performance deficit due to the brain injury. In order to cope with postinjury changes, caregivers are asked to “renegotiate relationships”, since the patient may often show a reduction in, if not a total lack of, emotional, intellectual, or financial contribution to the relationship because of the deficit following the sABI.

However, even if such role changes are distressing for most of caregivers, others express their satisfaction in supporting and assisting their loved one. According to Kosciulek [39], family adaptation to brain injury is defined as “the outcome of family efforts to bring a new level of balance, harmony, coherence, and a satisfactory level of functioning to a family following TBI”, and Verhaeghe and colleagues [15] underlined that a better recovery is more likely when caregivers cope effectively with the TBI.

The preinjury family dynamics and the caregivers' ability to access community resources [40], as well as* coping strategies, *such as acquisition of social support and resources, positive appraisal, and family tension management (e.g., sharing problems with other family members and taking a break from the care of patient) [39], seem to be related to better outcome in caregivers. Furthermore, “emotion focused strategies”, including acceptance, positive reappraisal, or seeking spiritual support, are positively linked to higher satisfaction of caregivers [41].

However, coping strategies of caregivers are strictly related to their level of burden, distress, and needs which, in turn, depend on the specific postinjury phase [42, 43]. Indeed,* family needs* may fluctuate and change over time [44]: in the early phase (e.g., acute care and postacute rehabilitation) the main caregivers' need is obtaining medical information on the patient, while a personal emotional support is required later, when they are no longer focused only on the patient's necessities [45]. Rotondi and colleagues [44] showed a modification of family needs throughout four phases, including acute phase, inpatient rehabilitation, the return to home, and post-return home (i.e., living in the community). Understanding type, consequences, and treatments of injury is the only common caregiver's need to these four phases. On the other hand, obtaining support from health professionals, family, and friends and being involved in the rehabilitation program are the major needs in the postacute rehabilitation and the return to home, while the necessity to manage and plan their own life is typical of the living in the community phase [44].

As a result of providing care, caregivers of sABI patients may also show* changes in their lifestyles* and, according to our substantial clinical expertise, a significant reduction of time spent in leisure or social activities may be associated with an increased risk for developing health problems or psychological distress. Indeed, as mentioned above, during the first postacute phase caregivers do not want to take time off from their daily caregiving activities and they are exclusively focused on their loved one: they are greatly involved in the caring process, losing the dimension of their* Self,* and reducing drastically their interests, hobbies, or activities related to social life [46]. However, literature to date is sparse about how changes in caregivers lifestyle may be related to the presence of psychological or emotional distress, whose high levels are associated with psychiatric disorders, such as depression and anxiety [47].

Furthermore, very little is known about questionnaires exploring lifestyle changes in caregivers of sABI patients, even though some scales assessing life changes as a result of caregiving exist in wider clinical population. Among these, the revised 15-item Bakas Caregiving Outcomes Scale (BCOS) [48] measures changes in time for family and social activities, emotional well-being, ability to cope with stress, level of energy, physical functioning, and general health.

In summary, since the caregivers' needs and lifestyles change over time, it is very important to adjust the psychological intervention on the basis of the specific caregiver situation. Indeed, there is no gold standard yet regarding the best approach to supporting sABI patients' caregivers in every setting, even though approaches including more interventions (e.g., educational methods, problem-solving techniques, and psychological support) [49], as well as online psychoeducational support groups [50], seem to better take into account the individuality of caregivers instead of choosing a single intervention [51], improving the family functioning.

Family support is fundamental from the early stages of the patient's hospitalization, to bear the physical, social, and financial costs of the rehabilitation [50] and improve QoL of both sABI patients and their caregivers. However, since the specific changes in lifestyle after the onset of a sABI has been poorly investigated in the literature, it is important to explore how the caregivers' lifestyle changes in relation to the type and amount of assistance to the sABI patients.

Aim of the study was to explore lifestyle changes of caregivers of individuals with sABI during the postacute rehabilitation phase, by investigating possible differences between primary and secondary caregivers.

Secondary aim was to investigate the possible important needs expressed by both primary and secondary caregivers, in order to identify the best psychological intervention to support them during the postacute rehabilitation phase.

## 2. Methods

### 2.1. Participants

By a cross-sectional study, 422 caregivers of 236 patients with sABI, consecutively admitted to the Post-Coma Unit of the IRCCS Fondazione Santa Lucia, a Postacute Rehabilitation Hospital in Rome (Italy), were enrolled in this study according to the following* inclusion* criteria: (1) age ≥ 18 years; (2) caregiver status of a sABI patient, diagnosed by a Glasgow Coma Scale (GCS) (Teasdale & Jennett 1974) score ≤ 8 in the acute phase. The only* exclusion* criterion was the presence or history of psychiatric disease in the caregiver.

After enrollment, 347 caregivers (M=41.9%, F 58.1%; mean age: 49.4 years, SD ±14.9) of 198 patients with sABI (M=56.1%, F= 43.9%; mean age: 44.2 years, SD ±17.0) provided their written informed consent to participate in this study.

The study was approved by the local Ethical Committee.

### 2.2. Measures

#### 2.2.1. Lifestyle Changes Questionnaire for Caregivers of Severe Acquired Brain Injury Patients (LCQ)

The literature about Scale assessing life changes in caregivers of sABI patients is still lacking. Accordingly, based on our clinical experience, an Italian unpublished 35-item self-report questionnaire has been developed by some authors of this study (E.A., U.B., M.D., D.S., and F.V.) with the purpose of exploring some lifestyles and needs of the caregivers of patients with sABI, during the postacute rehabilitation phase (see Appendix for the English version of the LCQ), in order to investigate the impact of sABI on the caregivers daily functioning, to better understand their burden, and, from a broader perspective, to help clinicians in improving their QoL.

The LCQ consists of 35 items and provides both multiple choice and some open-ended questions, gathering the caregivers' ideas or criticisms, which can be useful to the rehabilitation staff to support them, as effectively as possible. It provides sociodemographic and financial information (i.e., gender, age, educational level, profession, presence of financial problems related to the patient clinical condition, and kind of relationship with the patient) and some patients' clinical variables: injury aetiology (traumatic or nontraumatic), time since injury (chronicity), and type of admission (inpatient or outpatient).

Seven items (i.e., “time spent with friends”, “attendance at meeting places”, “reading books or newspapers”, “watching TV or listening to the radio”, “walking or riding bike”, “hobbies”, and “going to the cinema/theatre”) measure the* leisure activities* both in terms of frequency (“regularly”, “often”, or “never”) and in comparison to the pre-ABI condition (“more than before”, “as before”, or “less than before”); two items explore the* concrete or instrumental support* received by caregivers (“Are there people helping you in taking care of the patient?” and “Are there people helping you in managing daily activities, such as paying the bills?”); two items investigate their perceived or required* emotional support* (“Do you feel emotionally supported by your friends?” and “Have you ever received a psychological support?).

Further information about the* reason of assistance*,* spirituality* (“Has your faith always helped you to overcome the difficult moments of your life?”), and the* use of antidepressant or anxiolytic drugs* before and/or after patient's sABI onset was collected. Finally, the last part of the LCQ investigates some* possible needs and beliefs on the usefulness and benefit of receiving a psychological support*,* having group meetings with other family members* (in presence of a psychologist),* having exchange of information with other health professionals* (i.e., physician, nurses, speech therapists, physiotherapists, etc.), and* participating in mutual-support groups*. Caregivers were asked to describe both the positive and negative aspects of each of the above-mentioned activities (which they may have already experienced or would like to have) and to give their suggestions in order to help the rehabilitation team in improving the QoL of both sABI patients and their caregivers.

In summary, the LCQ was developed according to open interviews to the caregivers of patients with sABI during the last 5 years. We chose to include only the lifestyle domains (i.e., social activities) considered as the most affected ones, according to the point of view and the experience reported by our caregivers during the clinical interviews.

### 2.3. Procedure

Participants were recruited one week after admission of the sABI patients to the Post-Coma Unit, in order to give them enough time to experience the new situation of caregiver in the Neurorehabilitation Hospital. Around three weeks after the recruitment, the questionnaire was completed by the 347 enrolled caregivers by themselves, in the presence of a psychologist who assisted them in case of any doubt.

## 3. Data Analysis

Together with the assessment of the change of each leisure activity, an overall change in* leisure activities* after the onset of sABI (in comparison to the pre-sABI condition) was measured by summing the scores obtained on each item, rated according to a three-step Likert scale: +1: more than before, 0; as before, and -1: less than before. Accordingly, the leisure activities total score, named Overall Change Activities Score, was ranging between -7 (all the leisure activities are reduced) and +7 (all the leisure activities are improved).

In order to evaluate the support need in daily activities as well as in the emotional sphere, 2 scores were computed to evaluate the* total concrete support* and the* total emotional support*. All these scores were analyzed as ordinal scores according to the nonparametric approach, taking into account the fact that the data did not meet the assumptions of the parametric test, especially the assumption about normality of the distribution (scores show strong skewness).

Differences in categorical variables between respective comparison groups were analyzed using the Chi Square test, Fisher's exact test, or Kruskall-Wallis *χ*^2^ for equality-of-medians rank test.

The continuous variables were analyzed using Student's t-test, when applicable. Data analysis was performed using Stata/SE 13.1 (StataCorp, College Station, Texas, USA).

## 4. Results

In order to label caregivers on the basis of their involvement in caring (i.e., primary versus secondary caregivers), a preliminary investigation on the total amount of time (at least 12 hours/day) and activities (i.e., patients' eating, dressing, bathing, toileting, incontinence management, moving from bed to chair and vice versa, etc.) spent in caring of the patients allowed splitting 347 participants into 263 (75.8%) primary and 84 (24.2%) secondary caregivers, respectively. Their mean ages were 50.6 (SD±14.9) for primary and 45.7 years (SD±14.3) for secondary caregivers, and resulted to be significantly different (p<0.01). The issue about how much time the caregivers provided daily care was investigated by the LCQ, as highlighted by the question “How much time do you spend at the rehabilitation hospital?” (see Appendix).

Sociodemographic characteristics of caregivers and sABI patients are shown in Tables 1 and 2, respectively.

As for the time spent in informal caregiving, a statistically significant difference was found between groups of caregivers (p<0.01), while no differences were found between primary and secondary caregivers about working and financial consequences related to caregiving, even though almost half of the primary caregivers had financial problems (47,1%), probably due to the fact that they stopped working temporarily to provide care to their loved one (37,4%) (*see *Table 3).

About the reason of assistance, caregivers chose to take care of the sABI patients because “others people have no time” (overall: 49.8%, primary: 51.2%, and secondary: 42.8%, p=ns), “there is no one else who can do it in my place” (overall: 34.8%, primary: 35.0%, and secondary: 34.7%, p=ns), “I am more suitable than others” (overall: 14.6%, primary: 17.2%, and secondary: 4.1%, p<0.05), and “I have more time than other family members” (overall: 8.7%, primary: 7.4%, and secondary: 14.3%, p=ns).

Table 4 shows the percentage of primary and secondary caregivers* who modified their habits or leisure activities*, after the sABI. A significant difference between groups was found only in “attending meeting places” (p<0.01) and “having hobbies or interests” (p<0.05), revealing that the secondary caregivers showed a greater capability in maintaining their habits or leisure activities compared to primary caregivers. Moreover, 10.8% of the caregivers reported current use of antidepressant or anxiolytic drugs (primary 10.0%, secondary 13.6%, p=ns); interestingly, there was no history of psychoactive drug use before the ABI onset for two-thirds of them.

In examining the percentage of informal caregivers of sABI patients who have* never* been engaged in leisure activities after the patient's brain injury, we found statistically significant differences between the two groups of caregivers in “attending friends” and “going to the cinema or theatre”, showing that the percentage of primary caregivers who did not engage in these two activities was significantly higher than that of secondary caregivers (17, 9% versus 8,8% and 59,9% versus 47,0%, respectively). No other significant differences were found.

**Figure 1:**
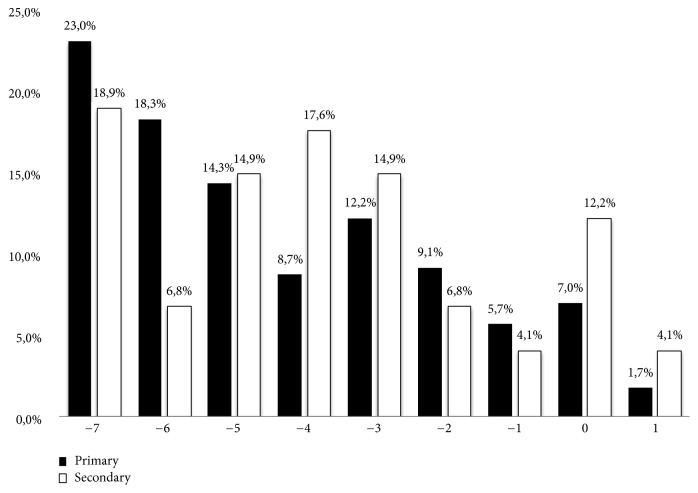
Primary and secondary caregivers' Overall Change Activities Score (from -7: “all the leisure activities have been reduced” to +7: “all the leisure activities have been improved”).

As shown in Figure 1, primary caregivers showed a statistically significant lower Overall Change Activities Score (i.e., reduced leisure activities) in comparison to the secondary caregivers (p<0.05). In fact, taking into account the left tail of the distribution (scores between -7 and -6), the percentage of primary caregivers with all reduced leisure activities was statistically significantly different from that of secondary caregivers (41,3% versus 25,7%; p<0.05).

Among the different needs included in the LCQ, no statistically significant differences were found between the two groups of caregivers. In particular, the most important need expressed by primary and secondary caregivers in the multiple answer questions was “having more medical information by health professionals” (68.1% and 64.3%, respectively), followed by “individual interview with a psychologist” (25.5% and 32.1%, respectively).

Taking into account the answers to open-ended questions, caregivers also expressed the need of being involved in the rehabilitation program, receiving a more emphatic communication by health professionals, getting better services.

The two groups differed statistically significantly in the total* concrete* support score (higher need of practical support, e.g., management of daily activities, showed by primary caregivers; Kruskal-Wallis *χ*^2^ = 4.941, p<0.05) while, interestingly, no difference was found in the total* emotional* support score. As for the spiritual support, 78.1% of the caregivers reported being helped by faith in overcoming the difficult moments of the life (primary: 79.6%, secondary: 72.7%, p=ns).

“Exchange of experiences” (primary: 39.9%, secondary: 34.5%, p=ns), the opportunity to “clarify doubts with experts” (primary: 37.6%, secondary: 38.1%, p=ns), and the opportunity to “understand my mistakes” (primary: 26.2%, secondary: 28.6%, p=ns) were considered the most important benefits revealed by the caregivers of taking part in mutual-support groups during the rehabilitation phase. For both groups, there were no drawbacks in participating in mutual-support groups (primary 62.4% versus secondary 60.6%, p=ns ), even though the embarrassment of speaking in public (primary 17.1% versus secondary 13.6%, p=ns) and about own personal issues (primary 14.4% versus secondary 16.7%, p=ns) and the fear of being depressed by listening to the other people's experiences (primary 16.0% versus secondary 16.7%, p=ns) were rated as possible limitations. Finally, a greater self-awareness and self-improvement were considered other probable advantages of participating to mutual-support groups.

## 5. Discussion

This study mainly aimed at exploring the possible lifestyle changes in a cohort of caregivers of individuals with sABI during the postacute rehabilitation phase, and in particular to investigate the variables of interest on the basis of the caregivers status (primary versus secondary).

Previous studies have mainly focused the attention on assessing family needs and conceptualizing the impact of sABI, especially TBI, on the family system which changes in its functionality, balance, and dynamics [25, 49, 52, 53], but very little is known in literature on how brain injury may have a negative impact on caregivers' lifestyle, often resulting in the loss of leisure activities, hobbies, and taking care of own appearance.

The present study tended to overcome limits of the existing literature, both by including a large sample of informal caregivers (primary and secondary) of individuals with sABI and by using the newly developed questionnaire LCQ to investigate their possible lifestyle changes (i.e., changes in social activities) as a result of caregiving.

In our study, primary caregivers most involved in the patient care were mainly parents and spouses, while siblings and offspring were the most important secondary caregivers.

In line with the literature on other clinical populations such as dementia [54], our results showed that after the sABI onset of their loved one,* primary *caregivers, compared to* secondary* caregivers, have never attended friends gatherings or gone to the cinema or theatre. This result may be related to the impossibility of staying physically away from their loved patients. In fact, sABI caregivers may have this kind of difficulty since they can feel guilty about dedicating time to themselves instead of their loved ones [55], and it can be associated with caregivers' distress, specifically depressive symptoms, anxiety, and burden, and with low levels of caregivers' frequency of leisure activities [55]. However, the role of the combination of guilt and frequency of leisure activities on caregivers' depressive symptoms is still controversial: it is plausible that caregivers with low frequency of leisure activities are the most prone to showing and maintaining depressive symptoms, but it is also reasonable that caregivers with high levels of leisure activities could present severe depressive symptoms, as a result of a moral conflict about the importance of maintaining leisure activities for themselves but simultaneously devoting time to caring for the patient [54].

Our data also showed that some other activities (i.e., reading books/newspapers or listening to the radio) have been maintained regardless of the role of the caregiving (primary or secondary). On the basis of our clinical expertise, we hypothesized that these leisure activities are compatible with the need and/or necessity of staying close to them.

By comparing the percentage of leisure activities performed by caregivers* before* and* after *the patient's sABI onset, we obtained that all caregivers showed high percentages of changes in lifestyle and habits, with a significant difference between groups in attending meeting places and having hobbies or interests. Also, in this case, the most affected leisure activities are those implying the “distance” from the patient. However, since caregivers of sABI patients often report financial consequences of caregiving [56], we also hypothesized that economic difficulties could have an impact on leisure activities participation, forcing caregivers to mainly sacrifice those implying specific costs (e.g., hobbies like going to the gym or swimming pool, etc.).

Our results reveal that primary caregivers reported more negative lifestyle changes than secondary caregivers, because of providing care for their loved one. The 23% of primary caregivers referred that all the 7 leisure activities explored by the LCQ were worsened after the sABI onset (as explained by Overall Change Activities Score equal to -7) in comparison to the 18,9% of secondary caregivers. More specifically, taking into account the left tail of the distribution showed in Figure 1 (scores between -7 and -6), the percentage of primary caregivers* with all or almost all* reduced leisure activities rose up to 41,3% compared to 25,7% of secondary caregivers. This finding may be related to the time spent in caregiving of sABI patients since, among the primary carers group, about 8% reported to provide care for their loved one for 24 hours* per day* or “all the time”, while 35.5% reported “all the day, but not night”. The percentage of primary caregivers daily involved in the sABI patient management is greatly higher than that of secondary caregivers and, accordingly, primary caregivers have not enough time for themselves and friends and lack more time for social and recreational activities.

However, our results also show that no statistically significant differences were found between primary and nonprimary caregivers about working and financial consequences related to caregiving, since both the two caregivers groups stopped working temporarily to provide care for their loved one, resulting in financial problems. These findings point out that, in terms of working and financial problems, secondary caregivers are affected to the same degree as primary ones.

In line with the existing literature regarding the emotional burden of being involved as a caregiver after a sABI [11, 12], our study demonstrates that being caregivers of sABI patients is demanding and that it may compromise the participation in leisure and recreational activities and, more in general, their QoL. In fact, our findings indicate that both primary and secondary carers modified their habits after the patient's brain injury with percentages rising up to 73,7%, as in the case of primary caregivers who do not attend friends gatherings as before. It could be reasonable to assume that primary caregivers experience more consequences, such as lack of time for social relations or physical problems, than secondary carers, but regarding the majority of caregiving consequences (i.e., financial problems or some leisure activities explored by LCQ) nonprimary caregivers are affected to the same degree as the primary ones, even though further investigations are needed to confirm this hypothesis.

As for the caregivers needs, they may be mediated through a complex network of characteristics, including gender, the caregiver-care recipient relationship, and employment status, even though, as mentioned above, caregiver needs change over time, as the caregiver moves through the care trajectory [57]. Our results showed that the most important need expressed by caregivers, during the postacute phase, was having more medical information by health professionals, followed by the need of being involved in the rehabilitation program. Primary and secondary caregivers only differed in the need for practical support (e.g., management of daily activities), suggesting that, obviously, primary caregivers are those mostly involved in the complex and stressful caregiving role, providing the great assistance, including personal care and household, financial, and emotional support [58].

One of the paradoxes in providing caregiver support is that, despite the caregivers' obvious needs, not all the available interventions are necessarily used [59, 60]. Thus, identifying the most important caregivers needs and understanding how they change over time is essential to supporting them in an appropriate way during each phase of their caregiving experience.

Since the knowledge about lifestyle changes in caregivers of individuals with sABI is still lacking, the strength point of this study is to give an overview about this issue: indeed, some studies revealed that the possible changes in caregivers lifestyle (mainly related to leisure activities) can have a meaningful impact on their psychological health, by triggering or increasing depressive symptoms [24]. Thus, our goal was to help psychologists and clinicians detect early indications of maladaptative lifestyle changes in caregivers of sABI patients and to identify priority areas for psychological interventions designed to improve caregivers' outcomes. Furthermore, the correlation between the lifestyles changes of the caregivers of individuals with sABI with the total amount of time and activities spent in caregiving role is a novel finding.

However, the present study shows some limits. Firstly, we used unvalidated and unpublished questionnaire, which could be a valuable measure in research to assess life changes but does not incorporate all the possible lifestyle domains, since it was developed by the authors in agreement with their experience in treating the caregivers during postacute rehabilitation phase. Another weak point of the study is the lack of investigation about the possible correlation between the caregivers lifestyle changes and the patients' level of disability (i.e., their neuropsychological and behavioral profiles).

Despite these limitations, our study underlines the usefulness of a psychological support to the caregivers, in order to modulate the time spent in the caregiving, and avoid the social isolation of the whole family.

Future studies are needed to evaluate the association between lifestyle changes due to caregiving and the risk of developing psychiatric disorders (i.e., anxiety and depression) in family members of sABI patients. Further studies are also warranted to investigate and identify needs, burden, and distress experienced by primary and secondary caregivers of individuals with sABI during the postacute rehabilitation phase, also in relation to the patients' outcome, in order to better address support interventions for them and improve their psychosocial outcomes and QoL.

However, the present study tried to provide some useful suggestions regarding the knowledge of the caregiver system in a specific milieu such as the postacute rehabilitation hospital and, accordingly, to help neuropsychologists and clinical psychologists to offer to caregivers a better psychological support on the basis of their real changes in lifestyle and needs.

## Figures and Tables

**Table 1:** Socio-demographic characteristics of caregivers.

**Characteristics**	**Primary caregivers**	**Secondary caregivers**	**Test**	**P**
***Mean age (years)***	50.6	45.7	t = -2.6001	**p<0.01**

***Gender***				
Male	111	35	*χ* ^2^ = 0.0000	n.s.
	*(42.2%)*	*(42.2%)*		
Female	152	48		
	*(57.8%)*	*(57.8%)*		

***Education***				
Primary school	14	3	*χ* ^2^ = 15.3470	**p<0.01**
	*(5.3%)*	*(3.6%)*		
Lower secondary school	62	11		
	*(23.7%)*	*(13.1%)*		
High school	137	40		
	*(52.3%)*	*(47.6%)*		
Bachelor's degree	20	7		
	*(7.6%)*	*(8.3%)*		
Master's degree	29	23		
	*(11.1%)*	*(27.4%)*		

***Profession***				
Full-time job	121	56	*χ* ^2^ = 12.4224	**p<0.05**
	*(46.0%)*	*(66.6%)*		
Unemployed	19	4		
	*(7.2%)*	*(4.8%)*		
Student	12	4		
	*(4.6%)*	*(4.8%)*		
Retired	59	12		
	*(22.4%)*	*(14.2%)*		
Housewife	35	4		
	*(13.3%)*	*(4.8%)*		
Other	17	4		
	*(6.5%)*	*(4.8%)*		

***Relationship***				
Mother	60	13	*χ* ^2^ = 16.5101	**p<0.05**
	*(26.0%)*	*(15.7%)*		
Father	33	11		
	*(12.6%)*	*(13.3%)*		
Siblings	31	19		
	*(11.8%)*	*(22.9%)*		
Wife	30	6		
	*(11.5%)*	*(7.2%)*		
Husband	29	5		
	*(11.1%)*	*(6.0%)*		
Cohabitant	14	2		
	*(5.3%)*	*(2.4%)*		
Offspring	40	22		
	*(15.3%)*	*(26.5%)*		
Other	17	5		
	*(6.5%)*	*(6.0%)*		

***Relationship as defined by caregivers***				
Very formal	3	3	*χ* ^2^ = 4.0529	n.s.
	*(1.1%)*	*(3.6%)*		
Formal enough	4	0		
	*(1.5%)*	*(0.0%)*		
Neither close, nor superficial	7	1		
	*(2.7%)*	*(1.2%)*		
Close enough	53	17		
	*(20.2%)*	*(20.2%)*		
Very close	196	63		
	*(74.5%)*	*(75.0%)*		

**Table 2:** Socio-demographic characteristics and clinical data of the sABI patients.

**Characteristics and clinical data**	**Inpatients**	**Outpatients**	**Test**	**P**
***Mean age (years)***	44.9	34.5	t = 2.1551	**p<0.05**

***Gender***				
Male	101	11	*χ* ^2^ = 4.5380	**p<0.05**
	*(54.3%)*	*(84.6%)*		
Female	85	2		
	*(45.7%)*	*(15.4%)*		

***Chronicity (days)***	111.7	421.5	t = -9.5264	**p<0.01**

***Aetiology***				
TBI	67	8	*χ* ^2^ = 3.3983	n.s.
	*(35.7%)*	*(61.5%)*		
non-TBI	77	3		
	*(41.6%)*	*(23.1%)*		
Other	42	2		
	*(22.7%)*	*(15.4%)*		

**Table 3:** Time spent in caregiving and related working and financial consequences.

	**Primary Caregivers**	**Secondary Caregivers**	**Test**	**p**
***Caregiving consequences***				
No consequence	149	58	*χ* ^2^ = 5.3920	n.s.
	*(58.7%)*	*(72.5%)*		
Work *temporarily* stopped	95	21		
	*(37.4%)*	*(26.3%)*		
Work *definitively* stopped	10	1		
	*(3.9%)*	*(1.2%)*		

Financial problems	123	33	*χ* ^2^ = 1.3792	n.s.
	*(47.1%)*	(39.8%)		

***Time spent in caregiving***				
Every day and night	20	1	*χ* ^2^ = 88.6949	**p<0.01**
	*(7.6%)*	*(1.2%)*		
All day, but not night	93	1		
	*(35.5%)*	*(1.2%)*		
Every half day	83	14		
	*(31.7%)*	*(16.7%)*		
Few hours a day	27	24		
	*(10.3%)*	*(28.6%)*		
Several hours a week	39	44		
	*(14.9%)*	*(52.4%)*		

**Table 4:** Caregivers who modified their habits or leisure activities after the sABI: comparison between primary and secondary caregivers.

**Leisure activities items**	**Primary** **Caregivers**	**Secondary** **Caregivers**	**Test**	**p**
Attending friends	191	55	*χ* ^2^ = 2.1383	ns
	*(73.7%)*	*(65.5%)*		
Attending meeting places (e.g. restaurant, bar, shopping centre, sport club, etc.)	174	46	*χ* ^2^ = 7.9014	**p<0.01**
	*(72.8%)*	*(56.1%)*		
Reading books/newspapers	132	39	*χ* ^2^ = 0.2904	ns
	*(50.4%)*	*(47.0%)*		
Watching TV/listening to the radio	159	42	*χ* ^2^ = 2.6361	ns
	*(60.7%)*	*(50.6%)*		
Walking or riding bike	155	41	*χ* ^2^ = 2.2197	ns
	*(62.7%)*	*(53.2%)*		
Having hobbies o interests	178	47	*χ* ^2^ = 4.0911	**p<0.05**
	*(71.2%)*	*(59.5%)*		
Going to the cinema/theatre	159	45	*χ* ^2^ = 2.0915	ns
	*(66.0%)*	(57.0%)		

## Data Availability

The authors recorded their data in an excel database, and it can be available in case of any need, upon submitting a request to the authors. However, it is not available online for the privacy of patient data.

## Appendix

### Lifestyle Changes and Needs Questionnaire for Caregivers of Severe Acquired Brain Injury Patients (LNQ)

**Cod.:**—

**Date of issue:** —/—/—

**Gender**

  □ Male
  □ Female

**Date of birth**

  —/—/—

**Educational level**

  □ Primary school
  □ Junior high school
  □ Secondary school
  □ Three-year degree
  □ Advanced degree
  □ Postgraduate specialisation
  □ Other (please specify) —

**Employment:**

  □ Pensioner
  □ Manager
  □ Unemployed
  □ Teacher
  □ Housewife
  □ Contractor
  □ Student
  □ Freelancer
  □ Workman
  □ Artisan/Trader
  □ Clerk
  □ Other —

**Did you suspend work to assist the patient?**

  □ Yes, definitely
  □ Yes, temporarily
  □ No

**Does the disease cause economic problems to your family?**

  □ Yes
  □ No

**Degree of relationship to the patient?**

  □ Mother
  □ Father
  □ Spouse
  □ Cohabiting/Partner
  □ Son/Daughter
  □ Brother/Sister
  □ Grandfather/Grandmother
  □ Uncle/Aunt
  □ Nephew
  □ Friend
  □ Legal guardian
  □ Other —

**What is the current hospitalization regime (please mark one answer)?**

  □ Inpatient, from (month and year): —
  □ Day Hospital, from (month and year): —
  □ Outpatient, from (month and year): —

**Why has the patient been admitted in this ward?**

  □ Traumatic brain injury
  □ Stroke
  Other (please specify): —

**Date of acquired brain injury**

  —/—/—

**How would you define your relationship with the patient?**

  □ Very formal
  □ Pretty formal
  □ Neither intimate, nor formal
  □ Pretty intimate
  □ Very intimate

**Why do you take care of patient? (one or more possible answers):**

  □ No one else can do it in my place
  □ I have more time than other family members
  □ Others do not have time
  □ I am more suitable than others
  other: —

**Does anyone help you in caring with the patient?**

  □ Yes
  □ No

**How much time do you spend at the rehabilitation hospital?**

  □ Twenty-four hours
  □ All the day (but not the night)
  □ Half a day
  □ A few hours a day
  □ Several hours a week (but not every day)
  □ Around 1-2 hours per week
  □ Only the weekend

**Does someone help you to manage your activities of daily living (eg, spending, paying bills, etc.)?**

  □ Yes
  □ No
  **Does one or more friends affectively support you?**
      □ No
      □ Yes, one
      □ Yes, many

**Do you hang out with friends?**

  □ More than once a week
  □ At least once a week
  □ Less than once a week
  □ Never
  **Compared to before the injury, you hang out them:**
      □ Less than before
      □ As before
      □ More than before

**Do you usually attend meeting places?**

  □ Regularly (every day)
  □ Often (more than once a week)
  □ Temporarily (less than once a week)
  □ Never
  **Compared to before the injury, you attend them:**
      □ Less than before
      □ As before
      □ More than before

**Do you read books/newspapers?**

  □ Regularly (every day)
  □ Often (more than once a week)
  □ Temporarily (less than once a week)
  □ Never
  **Compared to before the injury, you read:**
      □ Less than before
      □ As before
      □ More than before

**Do you watch TV or listen to the radio?**

  □ Regularly (every day)
  □ Often (more than once a week)
  □ Temporarily (less than once a week)
  □ Never
  **Compared to before the injury, you watch TV or listen to the radio:**
      □ Less than before
      □ As before
      □ More than before

**Do you walk or go biking?**

  □ Regularly (every day)
  □ Often (more than once a week)
  □ Temporarily (less than once a week)
  □ Never
  **Compared to before the injury, you walk or go biking:**
      □ Less than before
      □ As before
      □ More than before

**Do you have any hobbies?**

  □ Regularly (every day)
  □ Often (more than once a week)
  □ Temporarily (less than once a week)
  □ Never
  **Compared to before the injury, you enjoy your hobbies:**
      □ Less than before
      □ As before way
      □ More than before

**Do you go to the cinema or the theatre?**

  □ Regularly (every day)
  □ Often (more than once a week)
  □ Temporarily (less than once a week)
  □ Never
  **Compared to before the injury, you do it:**
      □ Less than before
      □ As before
      □ More than before

**Does faith help you in dealing with this difficult time?**

  □ Yes
  □ No

**Do you use psycho-drugs (e.g., for anxiety or depression)?**

  □ Yes, but already before the injury
  □ Yes, since the injury
  □ No

**What do you think are the most important needs of a caregiver?**

  □ Individual meetings with the psychologist
  □ Group meetings with other caregivers, assisted by a psychologist
  □ Talks or exchanges of information with other health professionals (e.g., physicians, nurses, speech therapists, physiotherapists, etc.)
  □ Recreational activities (e.g., music, movies, games, etc.)
  □ Other (please add any other information you think might be helpful):—

**Within our Rehabilitation Unit, what would you like that psychologists/therapists (or generally, healthcare staff) could do for you? (Please provide any information you think might be helpful).**

—

**Have you ever been supported (individually or in group) by a psychologist?**

  □ No
  □ Yes, individually
  □ Yes, in group
  □ Yes, both

**If so, why?**

  —
  **What were the positive aspects of that experience?** —
  **What were the negative aspects of that experience?** —

**If you had the opportunity to be included in a Self-Mutual-Support Group** **(The purpose of a Self-Mutual-** **Support Group is to give people in difficult situations** **an opportunity to share their experiences and help** **each other to manage common issues.)** **for the treatment of the family, to have you the possibility of listening and exchanging own experience with others, would you participate?**

  □ Yes
  □ No

**What do you think might be the benefits of this experience?**

  □ An exchange of experience
  □ A way to blow off some steam
  □ A possibility to spend time not only in caring the patient, but also to myself
  □ A space to better understand any mistakes made in caring the patient
  □ A possibility to be useful to other caregivers
  □ A possibility to understand how other caregivers have faced similar problems
  □ A possibility to clarify every doubts with professionals (psychologist, speech therapist, etc.)
  □ Other (please add any other information you think might be helpful): —

**What could be any negative aspects?**

  □ No one
  □ Fear of losing my “balance”
  □ Fear to weight me in listening to the experiences of others
  □ Fear of the judgment of others
  □ Embarrassment in speaking, in general, “in public”
  □ Embarrassment to talk to other people, specifically about my personal issues
  □ Other (Please add any other information you think might be helpful): —
